# Racial Differences in the Human Endogenous Circadian Period

**DOI:** 10.1371/journal.pone.0006014

**Published:** 2009-06-30

**Authors:** Mark R. Smith, Helen J. Burgess, Louis F. Fogg, Charmane I. Eastman

**Affiliations:** Biological Rhythms Research Laboratory, Department of Behavioral Sciences, Rush University Medical Center, Chicago, Illinois, United States of America; Vanderbilt University, United States of America

## Abstract

The length of the endogenous period of the human circadian clock (tau) is slightly greater than 24 hours. There are individual differences in tau, which influence the phase angle of entrainment to the light/dark (LD) cycle, and in doing so contribute to morningness-eveningness. We have recently reported that tau measured in subjects living on an ultradian LD cycle averaged 24.2 hours, and is similar to tau measured using different experimental methods. Here we report racial differences in tau. Subjects lived on an ultradian LD cycle (1.5 hours sleep, 2.5 hours wake) for 3 days. Circadian phase assessments were conducted before and after the ultradian days to determine the change in circadian phase, which was attributed to tau. African American subjects had a significantly shorter tau than subjects of other races. We also tested for racial differences in our previous circadian phase advancing and phase delaying studies. In the phase advancing study, subjects underwent 4 days of a gradually advancing sleep schedule combined with a bright light pulse upon awakening each morning. In the phase delaying study, subjects underwent 4 days of a gradually delaying sleep schedule combined with evening light pulses before bedtime. African American subjects had larger phase advances and smaller phase delays, relative to Caucasian subjects. The racial differences in tau and circadian phase shifting have important implications for understanding normal phase differences between individuals, for developing solutions to the problems of jet lag and shift work, and for the diagnosis and treatment of circadian rhythm based sleep disorders such as advanced and delayed sleep phase disorder.

## Introduction

Nearly all living organisms display circadian rhythms, which include a diverse array of near 24-hour cycles from the subcellular to the behavioral level. In sighted individuals the light/dark (LD) cycle is the main time cue that entrains circadian rhythms to the 24 hour day produced by the earth's rotation. In the absence of these time cues, circadian rhythms persist with an endogenous period (tau). There are inter-species differences in the length of tau, and within a species tau is normally distributed [1]. The average human tau is slightly greater than 24 hours [2], [3], [4], [5], [6], [7], [8]. Some of the differences in human tau have been attributed to age [9], [10] season [11], and sex [12], but no other factors have been identified as mediating the individual differences in human tau.

The phase angle of entrainment is the temporal interval between an output of the circadian clock [(e.g. the onset of wheel-running activity in a rodent, or in humans the time of the onset of melatonin secretion, marked by the dim light melatonin onset (DLMO)] and the LD cycle (e.g. lights on or sunrise). Tau influences the phase angle of entrainment, and thus is one factor that contributes to morningness-eveningness. For example, an animal with a longer tau begins its daily bout of activity at a relatively later time relative to the LD cycle than another animal of the same species with a shorter tau [13], [14], and could thus be thought of as more of a “night owl”. Duffy et al. [15] showed that a longer tau in humans is associated with a later habitual wake time, a later time of the minimum of the circadian rhythm of body temperature, and more eveningness on the Owl-Lark questionnaire [16]. Tau also influences the phase angle of entrainment between the endogenous circadian clock and the sleep schedule. In subjects with longer taus, the temporal interval between the DLMO and bedtime is shorter than in subjects with shorter taus [3], [17]. Thus subjects with longer taus (night owls or evening types) go to bed at an earlier circadian phase.

We have recently reported that human tau measured in an ultradian LD cycle averaged 24.2 hours [3], a free running period length very similar to previous findings. Here we report racial differences in tau. We also report racial differences in the magnitude of circadian phase shifts in response to bright-light pulses and a shift of the sleep/dark schedule.

## Methods

### Methods for Measuring Tau

Subjects (n = 60, 29 male, mean age±SD 26.33±5.48 years) maintained a regular sleep schedule at home for 1 week before coming to the laboratory for a 5-day session. The 5-day session included a baseline phase assessment, followed by 3 days of an ultradian light/dark cycle [3], [18], and then a final phase assessment (Figure S1). The ultradian light/dark cycle consisted of 1.5 hour episodes of darkness for sleep alternating with 2.5 hour episodes of wakefulness in dim room light [4,100°Kelvin (K), light exposure <100 lux]. Subjects completed two 5-day sessions, separated by one week. During one session they received a pill of exogenous melatonin or a bright light pulse on each of the 3 ultradian days, and during the other session they received placebo pills or no bright light, in counterbalanced order. Measurements of circadian period were calculated from the placebo or no bright light sessions only. The average circadian period for subjects that had the placebo 5-day session first (n = 33) or second (n = 27) was similar (24.25±0.23 and 24.24±0.21 hours, respectively). The individual differences in human tau have previously been ascribed to sex [12], age [9], [10], and season [11]. Data from two studies also suggests that iris color could influence sleep timing and circadian responses [19], [20]. Consequently, in addition to self-reported race as a predictor of tau, we included sex, age, month that tau was assessed, and iris color in a stepwise linear regression analysis.

To determine whether there were racial difference in the magnitude of the circadian phase shift in response to bright light exposure, we analyzed data from our recent phase advancing [21] and phase delaying [22] studies.

### Methods for the Phase Advancing Study

The protocol for the phase advancing study [21] is illustrated in Figure S2. Subjects maintained a regular sleep schedule for 10 days before coming into the laboratory for a baseline phase assessment. After the baseline phase assessment subjects resumed their regular sleep schedule at home for 11 days, during which time their baseline DLMO was determined. Subjects then slept in the laboratory for 4 treatment days. On the first treatment day they went to bed at their regular bedtime, were awakened 8 hours after their baseline DLMO, and were exposed to a 2-hour phase-advancing light pulse. Awakening time and the start time of the light pulse was advanced by 1 hour on each successive treatment day. Bedtime was also advanced so that the time in bed on the 2^nd^–4^th^ treatment nights was 8 hours. Following the 4 treatment days a final phase assessment was conducted to determine the time of the DLMO and assess the phase shift of the DLMO from the baseline to the final phase assessment. This was a between-subjects design in which subjects received light pulses from either polychromatic white (4,100°K; 6,000 lux; 4.9×10^15^ photons/cm^2^/sec) or blue-enriched (17,000°K; 4,000 lux; 4.2×10^15^ photons/cm^2^/sec) fluorescent lamps contained in a desk-top light box. Phase advances of the DLMO in response to the white and blue-enriched light pulses were similar, and the data from the two groups were combined for the current analysis of racial differences. Due to heterogeneity of variance, a Wilcoxon rank sum test was used to compare the phase advance of the DLMO in Caucasian (n = 10) and African American (n = 7) subjects.

### Methods for the Phase Delaying Study

The protocol for the phase delaying study is illustrated in Figure S3. The regular sleep schedule and phase assessments in the phase delaying study [22] were similar to the phase advancing study, but the light pulses and sleep episodes were timed to produce a circadian phase delay. On the first of 4 light treatment days subjects were exposed to a 2-hour light pulse, beginning 3 hours after their baseline DLMO. Following the light pulse subjects had 8 hours in bed in the dark. The time of the light pulse and the sleep episode were delayed 2 hours on each successive treatment day. This was a crossover design in which subjects were exposed to the same polychromatic white and blue-enriched light boxes as in the phase advancing study, in counterbalanced order, at equal photon density (4.2×10^15^ photons/cm^2^/sec). Phase delays in the two light conditions were very similar, and the average phase shift of the DLMO for the two conditions was used for the current analysis of racial differences. The magnitude of the phase delays of the DLMO for Caucasian (n = 9) and African American (n = 2) subjects are presented in the text, but because there were only 2 African American subjects we do not present a statistical test of their differences.

### Common Methods for Assessment of Circadian Phase

Details of phase assessments have been described previously [23]. During phase assessments subjects remained in dim light (4,100°K lamps covered with red filters, <3.8 µW/cm^2^) and provided saliva samples every 30 minutes. The concentration of melatonin in these saliva samples was determined by radioimmunoassay. The sensitivity of the assay was 0.7 pg/ml and the intra- and inter-assay coefficients of variability were 12.1% and 13.2%, respectively. Each melatonin profile was smoothed with a locally weighted least squares curve (GraphPad Prism, San Diego, CA). A threshold to determine the DLMO of each melatonin profile was calculated by taking the average of 5 consecutive low daytime values plus 2 standard deviations of these values [24]. The higher of the two thresholds (from the baseline or final melatonin profile) was applied to both profiles. The DLMO was defined as the time that the smoothed curve exceeded and remained above the threshold. In the phase advancing and phase delaying studies, the phase shift was calculated by taking the difference in the time of the DLMO between the baseline and final phase assessments. Because there were 4 days between the baseline and final phase assessments in the ultradian light/dark cycle, tau was calculated by dividing the phase shift of the DLMO by 4 and adding 24 hours. Measurements of circadian period, phase advances, and phase delays were also calculated using a different DLMO threshold [21], with similar results.

Protocols were approved by the Rush University Medical Center Institutional Review Board, and all subjects provided written informed consent before study participation commenced.

## Results

### Period Length (tau)

**Figure 1:**
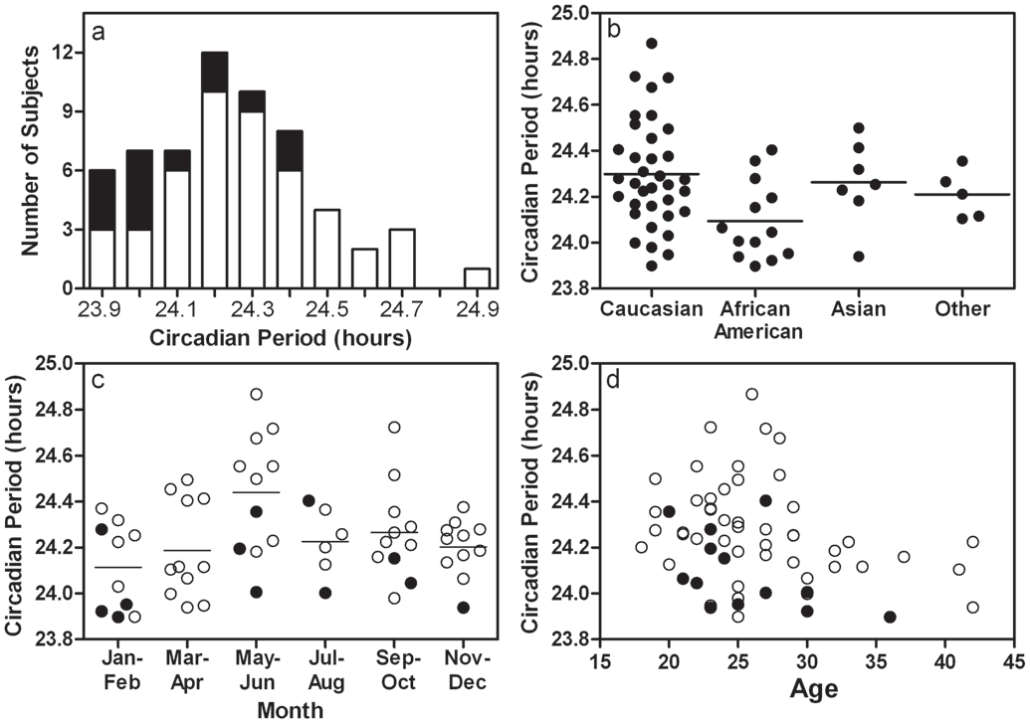
Racial differences in the human endogenous circadian period. The human endogenous circadian period depends on race, season, and age. (a) Histogram of circadian period (tau) for subjects (n = 60) whose self-reported race was African American (black bars) or not African American (white bars). (b) Circadian period by self-reported race. (c) Circadian period by 2 month bins. In (b) and (c), horizontal lines depict the mean. (d) Circadian period by age. Black dots in (c) and (d) indicate African American subjects.

The average circadian period was 24.24±0.22 (SD) hours (Fig. 1a). The stepwise linear regression analysis indicated that a model including race, month of assessment, and age was a significant predictor of tau [F(4,55) = 8.98, p<0.001]. Tau in African American subjects was significantly shorter (by 12.6 minutes) than for other subjects (Fig. 1a & b) [t = −3.85, p<0.001; unstandardized coefficient B = −0.21]. However, given the small number of Asian subjects, the only clear racial difference was between African American (24.09±0.17) and Caucasian (24.30±0.23 hours) subjects. Tau measured in May and June was significantly longer than in the other months, being lengthened by 12.5 and 16.8 minutes, respectively, relative to other months (Fig. 1c) [May: t = 2.86, p<0.01, unstandardized coefficient B = 0.21; June: t = 3.03, p<0.01, unstandardized coefficient B = 0.28]. Age was also a significant independent predictor of tau, such that older subjects had shorter taus (Fig. 1d) [t = −2.29, p = 0.03, unstandardized coefficient B = −0.01]. Although we enrolled a relatively narrow age range of subjects (18–45 years), all 13 of the subjects age 30 or older had a tau that was shorter than the group average, compared to 19 of 47 subjects younger than age 30 with a shorter than average tau (Fig. 1d) [χ^2^(1) = 14.52, p<0.001]. Tau was similar in females (24.20±0.19) and males (24.28±0.24 hours), and sex was not a significant independent predictor of tau.

African American race was the strongest predictor of tau (standardized coefficients β = −0.41), followed by measurement in June (β = 0.32), and May (β = 0.31), and age (β = −0.24). Together race, month of assessment, and age accounted for 40% of the variance in tau (R^2^ = 0.40).

### Phase Angle of Entrainment

In all subjects, tau was modestly associated with phase angle of entrainment, such that a longer tau was associated with a later DLMO relative to sunrise (r = 0.34, p<0.01). However, when the interval between the DLMO and bedtime was used as the phase angle of entrainment, the correlation with tau was in the predicted direction but did not reach statistical significance [r = −.23, p = 0.08].

In each of the 3 experiments described here (tau, phase-advancing, and phase-delaying), there were no racial differences in the bedtime, wake time, baseline DLMO, or the baseline DLMO to bedtime phase angle.

### Phase-Shifting Studies

**Figure 2:**
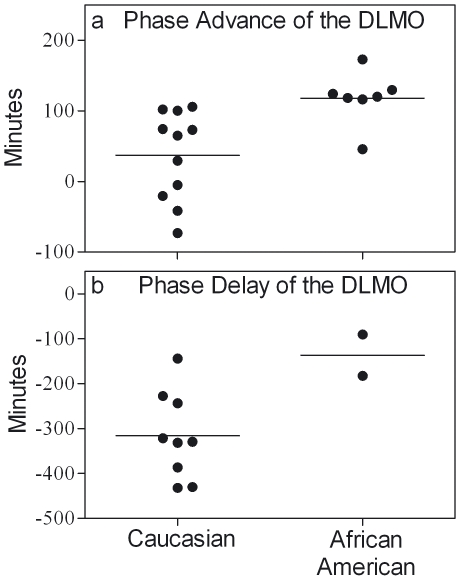
Racial differences in the magnitude of phase shifts to bright-light pulses and shifts of the sleep/dark schedule. Circadian phase advance (a) and phase delay (b) of the DLMO by self-reported race. By convention, phase advances are plotted as positive numbers and phase delays are plotted as negative numbers. Lines indicate the mean of each race.

In the phase-advancing study six of the seven African American subjects had larger phase advances than all 11 of the Caucasian subjects (Fig. 2a). The average phase advance for African American subjects (1.97±0.62 hours) was more than three times as large as for Caucasian subjects (0.56±1.09 hours) (Wilcoxon W = 60.00, Z = −2.93, p<0.01).

The average phase delay for African American subjects (−2.27±1.08 hours) was less than half as large as for Caucasian subjects (−5.27±1.61 hours) (Fig. 2b). It is prudent to interpret this difference with caution because of the small sample sizes.

### Iris Color

In the portion of our sample for which we have data on eye color, tau in subjects with brown (n = 29) and blue (n = 13) irises was similar (24.21±0.20 and 24.26±0.27 hours, respectively), and iris color was not a significant independent predictor of tau in the regression analysis.

In the phase advancing study [21] Caucasians with both light (blue and green) and dark (brown) irises were enrolled, as well as brown-eyed African Americans, enabling a comparison of the relative contribution of iris color versus race. While there was a difference in the phase advance of the DLMO based on race (described above), there was no significant difference in the phase advance between subjects with blue and green irises (n = 6) versus brown irises (n = 15) (1.1±1.2 and 1.3±1.2 hours, respectively). In the phase delaying study [22] we could not distinguish between iris color and race, since all the Caucasian subjects had light irises (blue, green, or hazel), and the two African American subjects had dark brown irises.

## Discussion

We have found that African Americans have a shorter free-running endogenous circadian period (tau) than Caucasians. This is the first report of racial differences in human tau. In addition, we present evidence that there are racial differences in the amount that the human circadian clock can be phase-shifted with bright light exposure and a shifted sleep/dark schedule, such that African Americans have larger phase advances, and smaller phase delays, relative to Caucasian subjects. Racial differences in childhood napping and nocturnal sleep as well as adult sleep architecture between African Americans and Caucasians have previously been reported [25], [26], [27], [28], [29], [30]. Whether and how these differences in sleep relate to differences in circadian rhythms have yet to be elucidated.

The racial differences we found in circadian phase shifting are consistent with what would be expected based solely on the differences in tau, not assuming any differences in the shape of the light phase response curves (PRCs). To illustrate why differences in tau would produce differences in the magnitude of phase shifts produced by our 4 day schedule of bright light pulses and shifting sleep/dark, consider these calculations: An individual with a tau of 24.1 hours (the African American average), if allowed to free-run, would delay 0.4 hours in 4 days, relative to the 24-hour LD cycle. Assuming that our stimuli advanced their free-running clock 0.5 hours per day (or 2.0 hours in 4 days), the net phase advance relative to the 24-hour LD cycle would be 2.0–0.4, or 1.6 hours. This is close to the actual average phase advance for our small sample of African American subjects, which was 2.0 hours. In contrast, an individual with a tau of 24.3 hours (the Caucasian average), if permitted to free-run, would delay 1.2 hours in 4 days. Assuming that our stimuli advanced his or her free-running clock the same 0.5 hours per day (or 2.0 in 4 days), the net phase advance relative to the 24-hour LD cycle would be 2.0–1.2, or 0.8 hours. This is also close to the actual average phase advance for our sample of Caucasians, which was 0.6 hours. Thus, based only on the differences in tau, African American subjects would have been expected to have larger phase advances and smaller phase delays than Caucasian subjects.

Daan & Pittendrigh [31] showed that animals with a short tau have a relatively enhanced delay zone of their light PRC, while those with a long tau have a relatively enhanced advance zone of their light PRC. These differences in PRC shape and amplitude presumably have adaptive significance in enabling animals with different length taus to entrain to the 24-hour LD cycle. We did not measure tau and circadian phase shifts in the same subjects, and it remains possible that humans with longer taus could also have a larger amplitude advance zone of their light PRC and advance more to the bright light stimulus than humans with shorter taus, who in turn could have a smaller amplitude phase advance zone of their light PRC.

We hypothesize that the racial differences in tau evolved because of latitude, with longer taus in the Caucasian population living at higher latitudes where there are greater seasonal changes in photoperiod, and shorter taus in African populations living closer to the equator where photoperiod is more constant. Latitudinal clines have been reported for the circadian rhythm of leaf movement in the flowering plant *Arabidopsis thalinia* [32] and the eclosion rhythm of the fruitfly *Drosophila auraria* [33], such that tau is longer at higher latitudes. In diurnal species with a tau >24 hours (e.g. humans), having a longer tau enhances the ability to track changes in photoperiod, which would be more prominent at higher latitudes, and to maintain the normal phase angle of entrainment between the circadian clock and the environmental LD cycle [14].

Weitzman et al. [9] reported that the period of the core body temperature rhythm in free-running older subjects (mean age 59.5) was shorter than in younger subjects (mean age 25.3). More recent data from a forced desynchrony protocol in which subjects lived on a 28 hour day failed to replicate this age-related shortening of tau [2]. We found that age was a significant independent but weak predictor of tau. Contrary to the hypothesis that tau shortens with age, a study in blind subjects reported that across a decade in mid-life (30–54 years), the length of tau in the same subjects increased with age [10]. Because our data and those described above [2], [9] are cross sectional, they do not exclude this latter possibility. An alternative explanation for the apparent discrepancy between our data and that of Kendall et al. [10] is that age-related changes in tau may be different for sighted and blind individuals.

We observed a longer tau in May and June than in other months. Similar seasonal effects in the period of core body temperature rhythm have been reported in subjects free-running in temporal isolation [11]. As stated by those authors, we do not know whether there is an actual annual rhythm in the endogenous circadian period, or whether the seasonal differences reflect aftereffects of a longer summer photoperiod on tau [34].

Higuchi et al. [20] reported that light-induced melatonin suppression in light-eyed Caucasian subjects was greater than in dark-eyed Asian subjects, but they could not distinguish between iris color and race. We did not observe differences in tau based on iris color. In our phase advancing study, in which iris color and race were not confounded, we observed differences in the phase advance between African Americans and Caucasians, but not between subjects with light or dark irises. Although these data suggest that race influences circadian phase shifts, this does not preclude a role of iris color as a contributing factor.

Our finding that tau was more strongly associated with the phase angle of entrainment to the natural LD cycle than to the behavioral LD cycle produced by the sleep/wake cycle is consistent with a previous report that the human circadian clock may entrain to sun time rather than clock time and the associated social time cues [35]. Although significant, the size of the correlation we found between tau and the phase angle of entrainment to sun time was modest, and we observed no racial differences in the phase angle of entrainment. One factor that could have reduced the strength of this association was the geographical location of our subject population (Chicago), since living in large cities (and presumably receiving less outdoor light exposure) has been shown to reduce the strength of the relationship between the natural LD cycle and the phase of the circadian clock [35].

In conclusion, we report racial differences in the endogenous period of the human circadian clock, with concomitant racial differences in circadian phase shifting. Because the period of the circadian clock influences the speed with which the circadian clock resets, our findings have broad implications for identifying the prevalence and improving the diagnosis and treatment of circadian-based sleep disorders, such as advanced and delayed sleep phase disorder, shiftwork disorder, and jet lag. For example, based upon the differences in tau, it is possible that the incidence of advanced sleep phase disorder is higher among African Americans, while the incidence of delayed sleep phase disorder is higher among Caucasians. Due to their shorter tau and larger phase advances, African American subjects might experience less jet lag when flying east, but more severe jet lag when flying west, compared to Caucasians. Finally, African Americans might show smaller phase delays during a night work and day sleep schedule, which could be associated with increased incidence of the deleterious consequences of circadian misalignment.

## Supporting Information

Figure S1 Protocol for assessing the endogenous circadian period. Subjects maintained a regular sleep schedule at home for at least one week before coming to the laboratory for a baseline phase assessment. This diagram shows the schedule for a subject that slept from 00:00–8:00 on days 1–7, but sleep schedules were tailored to each subject's habitual sleep times. The change in the time of the dim light melatonin onset (DLMO, indicated by the upward arrows) from the baseline to the final phase assessment was attributed to the free run of the endogenous circadian clock.
(0.06 MB PDF)

Figure S2 Protocol for the phase advancing study. Protocols were tailored to individuals' typical sleep schedules. This shows the protocol for a subject sleeping 00:00–8:00. The rectangle containing the “L” shows the time of the 2-hour bright light pulses. The first bright light pulse started 8 hours after the baseline DLMO, and the start time of the light pulses occurred one hour earlier on each successive day.
(0.07 MB PDF)

Figure S3 Protocol for the phase delaying study. Protocols were tailored to individuals' typical sleep schedules. This shows the protocol for a subject sleeping 00:00–8:00. The rectangle containing the “L” shows the time of the 2-hour bright light pulses. The first light pulse began 3 h after the baseline DLMO, and the start time of the light pulses occurred 2 h later on each successive day.
(0.05 MB PDF)
