# Supplementary figures and images for: Racial Differences in the Human Endogenous Circadian Period
Source: PLoS One. 2009 Jun 30;4(6):e6014. doi: 10.1371/journal.pone.0006014 (PMC2699031; doi:10.1371/journal.pone.0006014)

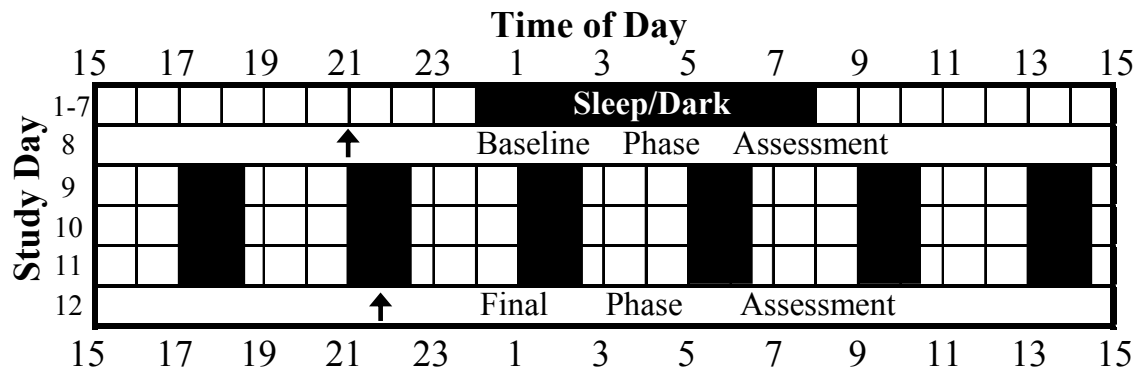

Supplement: Figure S1 — Protocol for assessing the endogenous circadian period. Subjects maintained a regular sleep schedule at home for at least one week before coming to the laboratory for a baseline phase assessment. This diagram shows the schedule for a subject that slept from 00:00–8:00 on days 1–7, but sleep schedules were tailored to each subject's habitual sleep times. The change in the time of the dim light melatonin onset (DLMO, indicated by the upward arrows) from the baseline to the final phase assessment was attributed to the free run of the endogenous circadian clock. (0.06 MB PDF) [file pone.0006014.s001.pdf]

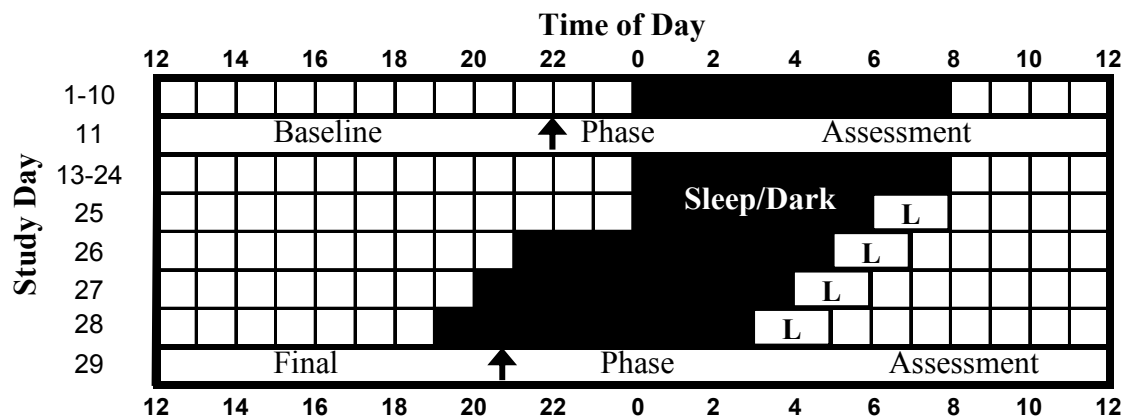

Supplement: Figure S2 — Protocol for the phase advancing study. Protocols were tailored to individuals' typical sleep schedules. This shows the protocol for a subject sleeping 00:00–8:00. The rectangle containing the “L” shows the time of the 2-hour bright light pulses. The first bright light pulse started 8 hours after the baseline DLMO, and the start time of the light pulses occurred one hour earlier on each successive day. (0.07 MB PDF) [file pone.0006014.s002.pdf]

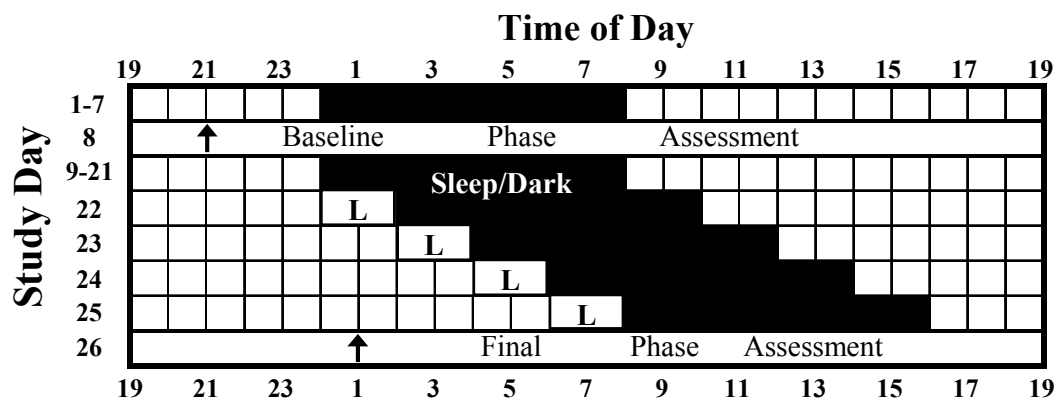

Supplement: Figure S3 — Protocol for the phase delaying study. Protocols were tailored to individuals' typical sleep schedules. This shows the protocol for a subject sleeping 00:00–8:00. The rectangle containing the “L” shows the time of the 2-hour bright light pulses. The first light pulse began 3 h after the baseline DLMO, and the start time of the light pulses occurred 2 h later on each successive day. (0.05 MB PDF) [file pone.0006014.s003.pdf]
